# Supplementary material for: Septins function in exocytosis via physical interactions with the exocyst complex in fission yeast cytokinesis
Source: bioRxiv. 2025 Jul 1:2024.07.09.602728. Originally published 2024 Jul 12. Preprint. [Version 2] doi: 10.1101/2024.07.09.602728 (PMC11257574; doi:10.1101/2024.07.09.602728)
Supplement: Supplement 1 [file NIHPP2024.07.09.602728v2-supplement-1.pdf]

bioRxiv preprint doi: https://doi.org/10.1101/2024.07.09.602728; this version posted July 1, 2025. The copyright holder for this preprint (which was not certified by peer review) is the author/funder, who has granted bioRxiv a license to display the preprint in perpetuity. It is made available under aCC-BY 4.0 International license.

**Additional files**

**Supplementary files**

Table S1. *S. pombe* strains used in this study.

Figure S1. Localization, levels, and dynamics of septin and exocyst subunits.

Figure S2. Localization of Sec3 and Spn1 in *gef3, rho4,* and *gef3 rho4* mutants.

Figure S3. AlphaFold predicted models of septin and exocyst subunit interactions.

Figure S4. The predicted 3D structural model of the exocyst complex by AlphaFold3.

Figure S5. The predicted 3D structural models of septin complexes by AlphaFold3.

Figure S6. Septin subunits interact with exocyst subunits in reciprocal Co-IPs.

Video 1. Accumulation of the septin Spn1-mEGFP and the exocyst Exo70-tdTomato to the division site.

Video 2. Dynamic localization of Exo70-tdTomato at the division site on a single-focal plane close to the cell surface.

Video 3. Dynamic localization of Exo70-tdTomato at the division site on the middle-focal plane.

Video 4. AlphaFold modeling of fission yeast exocyst complex.

Video 5. AlphaFold modeling of fission yeast septins (Spn1, 2, 3, and 4) octameric complex.

Video 6. AlphaFold modeling of septins Spn1, Spn2, Spn4 hexameric complex.

Video 7. AlphaFold modeling of septins Spn1, Spn3, Spn4 hexameric complex.

Video 8. Localization of Sec15-mEGFP in WT cells.

Video 9. The mislocalization of Sec15-mEGFP on the division plane in *spn1Δ* cells during late-stage of cytokinesis.

Video 10. The mislocalization of Sec15-mEGFP on the division plane in *spn4Δ* cells during late-stage of cytokinesis.

bioRxiv preprint doi: https://doi.org/10.1101/2024.07.09.602728; this version posted July 1, 2025. The copyright holder for this preprint (which was not certified by peer review) is the author/funder, who has granted bioRxiv a license to display the preprint in perpetuity. It is made available under aCC-BY 4.0 International license.

**Supplemental Table 1. *S. pombe* strains used in this study.**

| Strain | Genotype | Figure; Video; Table; (Reference) |
|---|---|---|
| JW8692 | *sec3-tdTomato-hphMX6 spn1-mEGFP-kanMX6 sad1-mRFP1-kanMX6 ade6-M210 leu1-32 ura4-D18* | Figure 1, A, B, D and E |
| JW9170 | *spn1-mEGFP-kanMX6 exo70-tdTomato-natMX6 ade6-M210 leu1-32 ura4-D18* | Figure 1C; Videos 1-3 |
| JW1113 | $h^-$ *spn1-mEGFP-kanMX6 sad1-mRFP1-kanMX6 ade6-M210 leu1-32 ura4-D18* | Figure 1F |
| JW1100 | *spn1-mEGFP-kanMX6 ade6-M210 leu1-32 ura4-D18* | Figures 1, H and I; 4C; S2B; S6A |
| JW8928 | *spn1-mEGFP-kanMX6 sec3-913-hphMX6 ade6 leu1-32 ura4-D18* | Figures 1, F, H and I; S1, A, C and E |
| JW8848 | *sec8-1 spn1-mEGFP-kanMX6 sad1-mRFP1-kanMX6 rng8-tdTomato-kanMX6 ade6-M210 leu1-32 ura4-D18* | Figure 1G |
| IJ612 | $h^+$ *sec3-GFP-kanMX6 ade6-M216 leu1-32 ura4-D18* | Figures 2, A-C; S1I; S2A; (Jourdain et al., 2012) |
| JW7322 | *spn1-Δ2::kanMX6 sec3-GFP-kanMX6 ade6-M216 leu1-32 ura4-D18* | Figures 2, A-C; S1I |
| JW7061 | $h^-$ *sec8-GFP-ura4*$^+$ *rlc1-tdTomato-natMX6 ade6? leu1-32 ura4-D18* | Figures 2, D and E; S1, G and H |
| JW8295 | *sec8-GFP-ura4*$^+$ *rlc1-tdTomato-natMX6 spn1-Δ2::kanMX6 leu1-32 ura4-D18* | Figures 2, D and E; S1, G and H |
| JW9737 | $h^+$ *spn2-mEGFP-kanMX6 ade6-M210 ura4-D18 leu1-32* | Figure 4A |
| JW9731 | $h^-$ *sec15-13Myc-kanMX6 ade6-210 ura4-D18 leu1-32* | Figures 4, A and C; S6E |
| JW9757 | *spn2-mEGFP-kanMX6 sec15-13Myc-kanMX6 ade6-M210 ura4-D18 leu1-32* | Figure 4A |
| JW9756 | $h^+$ *spn2-13Myc-kanMX6 ade6-M210 leu1-32 ura4-D18* | Figure 4B |
| JW9726 | $h^-$ *sec15-mEGFP-kanMX6 ade6-210 ura4-D18 leu1-32* | Figure 4, B and D; Video 8 |
| JW9771 | *spn2-13Myc-kanMX6 sec15-mEGFP-kanMX6 ade6-210 ura4-D18 leu1-32* | Figure 4B |
| JW9744 | $h^+$ *spn1-mEGFP-kanMX6 sec15-13Myc-kanMX6 ade6-M210 leu1-32 ura4-D18* | Figure 4C |
| JW9733 | $h^-$ *spn1-13Myc-hphMX6 sec15-mEGFP-kanMX6 ade6-M210 ura4-D18 leu1-32* | Figure 4D |
| JW8596 | $h^+$ *spn1-13Myc-hphMX6 ade6-M210 leu1-32 ura4-D18* | Figures 4D; S6B |

bioRxiv preprint doi: https://doi.org/10.1101/2024.07.09.602728; this version posted July 1, 2025. The copyright holder for this preprint (which was not certified by peer review) is the author/funder, who has granted bioRxiv a license to display the preprint in perpetuity. It is made available under aCC-BY 4.0 International license.

| | | |
|---|---|---|
| JW9789 | $h^+$ spn2-Δ1::hphMX6 sec15-mEGFP-kanMX6 ade6-210 ura4-D18 leu1-32 | Figure 5, A and B |
| JW9759 | $h^+$ sec15-mEGFP-kanMX6 ade6-210 ura4-D18 leu1-32 | Figures 5, A and B; S6F |
| JW9852 | sec15-mEGFP-kanMX6 spn1-Δ2::kanMX6 ade6-M210? ura4-D18 leu1-32 | Figure 5, A and B; Video 9 |
| JW9853 | sec15-mEGFP-kanMX6 spn4-Δ2::hphMX6 ade6-M210 ura4-D18 leu1-32 | Figure 5, A and B; Video 10 |
| JW9804 | spn2-Δ1::hphMX6 sec5-mEGFP-kanMX6 ade6-210 ura4-D18 leu1-32 | Figure 5, C and D |
| JW9791 | $h^+$ sec5-mEGFP-kanMX6 ade6-M210 ura4-D18 leu1-32 | Figure 5, C and D |
| JW81 | $h^-$ ade6-210 ura4-D18 leu1-32 | Figures 6, A and B; 7B |
| JW289 | $h^+$ spn1-Δ2::kanMX6 leu1-32 ura4-D18 | Figures 6, A and B; 7B; Table 1 |
| MBY887 | $h^+$ sec8-1 ura4-D18 leu1-32 | Figures 6, A and B; 7B; (Wang et al., 2002) |
| JW7130 | $h^-$ kanMX6-Pypt3-mEGFP-ypt3 ade6-210 leu1-32 ura4-D18 | Figure 6C |
| JW7354 | spn1-Δ2::kanMX6 kanMX6-Pypt3-mEGFP-ypt3 leu1-32 ura4-D18 | Figure 6C |
| JW6548 | $h^+$ GFP-syb1-kanMX6 rlc1-tdTomato-natMX6 ade6 leu1-32 ura4-D18 | Figure 6D |
| JW7385 | spn1-Δ2::kanMX6 GFP-syb1-kanMX6 rlc1-tdTomato-natMX6 leu1-32 ura4-D18 | Figure 6D |
| JW5249 | GFP-bgs1-leu1$^+$ bgs1Δ::ura4$^+$ rlc1-tdTomato-natMX6 ade6-M210 leu1-32 ura4-D18 | Figure 7A |
| JW7264 | GFP-bgs1-leu1$^+$ bgs1Δ::ura4$^+$ rlc1-tdTomato-natMX6 spn1-Δ2::kanMX6 ade6 ura4-D18 | Figure 7A |
| PPG37.23 | $h^-$ eng1-GFP-kan$^R$ leu1-32 ura4-D18 | Figure 7C (Santos et al., 2005) |
| JW9057 | eng1-GFP-kan$^R$ spn1-Δ2::kanMX6 leu1-32 ura4-D18 | Figure 7C |
| JW1113 | $h^-$ spn1-mEGFP-kanMX6 sad1-mRFP1-kanMX6 ade6-M210 leu1-32 ura4-D18 | Figure S1, A, C-E; |
| JW8829 | exo70Δ::kanMX4 spn1-mEGFP-kanMX6 sad1-mRFP1-kanMX6 ade6 leu1-32 ura4-D18 | Figure S1, A-D |
| JW8830 | sec8-1 spn1-mEGFP-kanMX6 sad1-mRFP1-kanMX6 ade6-M210 leu1-32 ura4-D18 | Figure S1, A-D |
| JW8929 | $h^-$ exo70-mEGFP-kanMX6 ade6-M210 ura4-D18 leu1-32 | Figure S1, F and H |
| JW8960 | exo70-mEGFP-kanMX6 spn1-Δ2::kanMX6 ade6-M210 leu1-32 ura4-D18 | Figure S1, F and H |

bioRxiv preprint doi: https://doi.org/10.1101/2024.07.09.602728; this version posted July 1, 2025. The copyright holder for this preprint (which was not certified by peer review) is the author/funder, who has granted bioRxiv a license to display the preprint in perpetuity. It is made available under aCC-BY 4.0 International license.

| | | |
|---|---|---|
| JW8938 | *rho4Δ::kanMX6 sec3-GFP-kanMX6 leu1-32 ura4-D18* | Figure S2A |
| JW8955 | *gef3Δ::hphMX6 sec3-GFP-kanMX6 ade6-M210 leu1-32 ura4-D18* | Figure S2A |
| JW8959 | *gef3Δ::hphMX6 rho4Δ::kanMX4 sec3-GFP-kanMX6 ade6? leu1-32? ura4-D18* | Figure S2A |
| JW9058 | *gef3Δ::hphMX6 rho4Δ::kanMX4 spn1-mEGFP-kanMX6 ade6 leu1-32 ura4-D18* | Figure S2B |
| JW9765 | $h^-$ *sec6-13Myc-kanMX6 ade6-210 ura4-D18 leu1-32* | Figure S6A |
| JW9774 | *spn1-mEGFP-kanMX6 sec6-13Myc-kanMX6 ade6-M210 leu1-32 ura4-D18* | Figure S6A |
| JW9766 | $h^-$ *sec6-mEGFP-kanMX6 ade6-210 ura4-D18 leu1-32* | Figure S6B |
| JW9832 | *spn1-13Myc-hphMX6 sec6-mEGFP-kanMX6 ade6-210 ura4-D18 leu1-32* | Figure S6B |
| JW8778 | $h^-$ *spn2-mEGFP-kanMX6 ade6-M210 leu1-32 ura4-D18* | Figure S6C |
| JW9755 | $h^+$ *sec5-13Myc-kanMX6 ade6-M210 leu1-32 ura4-D18* | Figure S6C |
| JW9772 | *sec5-13Myc-kanMX6 spn2-mEGFP-kanMX6 ade6-210 ura4-D18 leu1-32* | Figure S6C |
| JW9756 | $h^+$ *spn2-13Myc-kanMX6 ade6-M210 leu1-32 ura4-D18* | Figure S6D |
| JW9738 | $h^-$ *sec5-mEGFP-kanMX6 ade6-M210 ura4-D18 leu1-32* | Figure S6D |
| JW9775 | *spn2-13Myc-kanMX6 sec5-mEGFP-kanMX6 ade6-M210 leu1-32 ura4-D18* | Figure S6D |
| JW1171 | $h^+$ *spn4-mYFP-kanMX6 ade6-M210 leu1-32 ura4-D18* | Figure S6, E and G |
| JW9829 | *sec15-13Myc-kanMX6 spn4-mYFP-kanMX6 ade6-M210 ura4-D18 leu1-32* | Figure S6E |
| JW9854 | *spn4-13Myc-kanMX6 sec15-mEGFP-kanMX6 ade6-210 ura4-D18 leu1-32* | Figure S6F |
| JW9768 | $h^-$ *spn4-13Myc-kanMX6 ade6-210 ura4-D18 leu1-32* | Figure S6, F and H |
| JW9139 | $h^-$ *sec3-13Myc-natMX6 ade6-210 leu1-32 ura4-D18* | Figure S6G |
| JW9711 | *spn4-mYFP-kanMX6 sec3-13Myc-natMX6 ade6-M210 leu1-32 ura4-D18* | Figure S6G |
| JW7300 | $h^+$ *sec3-GFP-kanMX6 ade6-M210 leu1-32 ura4-D18* | Figure S6H |
| JW9788 | *spn4-13Myc-kanMX6 sec3-GFP-kanMX6 ade6-210 leu1-32 ura4-D18* | Figure S6H |

bioRxiv preprint doi: https://doi.org/10.1101/2024.07.09.602728; this version posted July 1, 2025. The copyright holder for this preprint (which was not certified by peer review) is the author/funder, who has granted bioRxiv a license to display the preprint in perpetuity. It is made available under aCC-BY 4.0 International license.

| | | |
|---|---|---|
| JW7035 | *h⁻ trs120-M1-his5⁺-kanMX6 his5Δ ade6-M210 leu1-32 ura4* | Tables 1 and 2 |
| JW8821 | *trs120-M1-his5⁺-kanMX6 spn1-Δ2::kanMX6 leu1-32 ura4* | Tables 1 and 2 |
| JW7036 | *h⁻ trs120-ts1-his5⁺-kanMX6 his5Δ ade6-M210 leu1-32 ura4* | Tables 1 and 2 |
| JW8822 | *trs120-ts1-his5⁺-kanMX6 spn1-Δ2::kanMX6 leu1-32 ura4* | Tables 1 and 2 |
| JW290 | *h⁻ spn1-Δ2::kanMX6 his3-27 ura4-D18* | Tables 1 and 2 |
| MBY887 | *h⁺ sec8-1 ura4-D18 leu1-32* | Tables 1 and 2 |
| JW8796 | *spn1-Δ2::kanMX6 sec8-1 ura4-D18* | Tables 1 and 2 |
| JW2716 | *h⁺ exo70Δ::kanMX4 ade6 leu1-32 ura4-D18* | Tables 1 and 2 |
| JW8797 | *spn1-Δ2::kanMX6 exo70Δ::kanMX4 his3-27 ade6 ura4-D18* | Tables 1 and 2 |
| IJ1032 | *h⁻ sec3-916-hphMX6 ade6-M216 leu1-32 ura4-D18* | Tables 1 and 2; (Jourdain et al., 2012) |
| JW8787 | *spn1-Δ2::kanMX6 sec3-916-hphMX6 ade6-M216 leu1-32 ura4-D18* | Tables 1 and 2 |
| IJ767 | *h⁻ sec3-913-hphMX6 ade6-M216 leu1-32 ura4-D18* | Tables 1 and 2; (Jourdain et al., 2012) |
| JW8783 | *spn1-Δ2::kanMX6 sec3-913-hphMX6 ade6-M216 leu1-32 ura4-D18* | Tables 1 and 2 |
| JW8588 | *spn2-Δ1::ura4⁺ ade6 leu1-32 ura4-D18* | Tables 1 and 2 |
| JW8782 | *spn2-Δ1::ura4⁺ sec3-913-hphMX6 ade6 leu1-32 ura4-D18* | Tables 1 and 2 |
| JW8789 | *spn2-Δ1::ura4⁺ sec3-916-hphMX6 ade6 leu1-32 ura4-D18* | Tables 1 and 2 |
| JW8590 | *h⁺ spn3-Δ2::kanMX6 ade6-M210 leu1-32 ura4-D18* | Tables 1 and 2 |
| JW8790 | *spn3-Δ2::kanMX6 sec3-913-hphMX6 ade6-M21X leu1-32 ura4-D18* | Tables 1 and 2 |
| JW8785 | *spn3-Δ2::kanMX6 sec3-916-hphMX6 ade6-M21X leu1-32 ura4-D18* | Tables 1 and 2 |
| JW293 | *h⁻ spn4-Δ2::kanMX6 ura4-D18* | Tables 1 and 2 |
| JW295 | *h⁺ spn4-Δ2::kanMX6 leu1-32 ura4-D18* | Tables 1 and 2 |
| JW8784 | *spn4-Δ2::kanMX6 sec3-913-hphMX6 ade6-M216 leu1-32 ura4-D18* | Tables 1 and 2 |
| JW8788 | *spn4-Δ2::kanMX6 sec3-916-hphMX6 leu1-32 ura4-D18* | Tables 1 and 2 |
| JW8799 | *spn4-Δ2::kanMX6 sec8-1 leu1-32 ura4-D18* | Tables 1 and 2 |

bioRxiv preprint doi: https://doi.org/10.1101/2024.07.09.602728; this version posted July 1, 2025. The copyright holder for this preprint (which was not certified by peer review) is the author/funder, who has granted bioRxiv a license to display the preprint in perpetuity. It is made available under aCC-BY 4.0 International license.

# Figure supplement legends

**Figure S1. Localization and division site levels of septin and exocyst subunits in mutants; and FRAP analyses of Spn1 and Sec3. (A and B)** Localization of Spn1 in WT and exocyst mutants at 25°C (A) and 4 h at 36°C (B). Arrows indicate cells with mislocalized Spn1 at the center of the division plane. **(C and D)** Quantifications of Spn1 intensities at the division site in WT and exocyst mutants at 25°C (C) and 4 h at 36°C (D). No septum: cells with Spn1 signal at the division site but no septum is visible under DIC; forming septum: septum with a visible gap in the middle; closed septum: no visible gap in the septum. *, $P < 0.05$; **, $P < 0.01$; ***, $P < 0.001$. **(E)** FRAP analyses of Spn1 at the division site in WT and *sec3-913* cells grown at 36°C for 4 h. Time-lapse images show recovery of Spn1 signals over time. Red box marks the region photobleached at time 0. **(F and G)** Localization of Exo70 (F) and Sec8 (G) in WT and *spn1*Δ cells. Yellow boxes, cells without a septum; Red boxes, cells with a closed septum. **(H)** Quantifications of Exo70 (left) and Sec8 (right) intensities at the division site in WT and *spn1*Δ cells. ***, $P < 0.001$. **(I)** FRAP analyses of Sec3 at the division site in WT and *spn1*Δ cells. Red box marks the region photobleached at time 0. Bars, 5 µm.

**Figure S2. Sec3 and Spn1 localization in *gef3*, *rho4,* or *gef3 rho4* mutants. (A)** Sec3 localization in WT, *rho4*Δ, *gef3*Δ, and *gef3*Δ *rho4*Δ cells. Arrowheads mark examples of the cells with mislocalized Sec3 at the center of the division plane in mutant but not WT cells. End-on views of the division plane of cells with a closed septum are shown on the last column. **(B)** Spn1 localization in WT and *gef3*Δ *rho4*Δ cells. Bars, 5 µm.

**Figure S3. The 3D structural models of septin-exocyst interactions generated by AlphaFold. (A, C, E, G, I)** Rank 1 model of AlphaFold2_advanced predicted interaction between Sec15 and Spn1 (A, pTM score = 0.47), Sec6 and Spn1 (C, pTM = 0.45), Spn2 and Sec5 (E, pTM = 0.37), Spn4 and Sec15 (G, pTM = 0.48), and Spn4 and Sec3 (I, pTM = 0.43). Septin subunits are colored in yellow and the exocyst in magenta, contacts between interface residues with distance < 4 Å are colored in cyan**. (B, D, F, H, J)** pLDDT scores of five predicted models and the PAE plot of rank1 model for Sec15 and Spn1 (B), Sec6 and Spn1 (D), Spn2 and Sec5 (F), Spn4 and Sec15 (H), and Spn4 and Sec3 (J).

**Figure S4. The predicted 3D structural model of *S. pombe* exocyst complex by AlphaFold3, highlighting the residues that interact with septins (also see Video 4).** Individual subunits are colored distinctly and labeled. Surface exposed residues previously identified as putative septin-interacting sites are highlighted in yellow. The model demonstrates that ~84% predicted septin-interacting residues are accessible on the outer surface of the assembled complex. As AlphaFold3 limits to fit the whole complex in 5000 tokens, full length Sec5, Exo70, Ex84 were used, but only amino acids 1-500 for Sec3, 1-546 for Sec6, 1-865 for Sec8, 1-620 for Sec10, and 1-396 for Sec15 were used. These truncations were selected based on the budding yeast exocyst cryo-EM structure (PDB: 5YFP), which shows that these regions are sufficient for stable inter-subunit interactions and are unlikely to interfere with septin binding based on our modeling.

**Figure S5. The predicted 3D structural models of *S. pombe* septin complexes by AlphaFold3, highlighting the exocyst-interacting residues (Videos 5-7). (A-C)** Two subunits of each septin were used to construct the octameric or hexameric complex. Different subunits are

bioRxiv preprint doi: https://doi.org/10.1101/2024.07.09.602728; this version posted July 1, 2025. The copyright holder for this preprint (which was not certified by peer review) is the author/funder, who has granted bioRxiv a license to display the preprint in perpetuity. It is made available under aCC-BY 4.0 International license.

colored distinctly and labeled. Surface exposed residues previously identified as putative exocyst-interacting sites are highlighted in yellow. (A) The octameric model of Spn1 to Spn4 demonstrates that ~96% predicted exocyst-interacting residues are accessible on the outer surface of the assembled complex. (B) The hexameric complex of two subunits of each Spn1, Spn2, and Spn4 shows 92%, (C) of each Spn1, Spn3 and Spn4 shows 86% exocyst-interacting residues are available on outer surface of assembled complex.

**Figure S6. Septins and the exocyst interact physically.** Reciprocal co-immunoprecipitation between Spn1 with Sec6 **(A, B)**; Spn2 with Sec5 **(C, D)**; Spn4 with Sec15 **(E, F)**; and Spn4 with Sec3 **(G, H)**. Septin or exocyst subunits tagged with mEGFP, GFP, mYFP, or 13Myc were immunoprecipitated, separated on SDS-PAGE, and incubated with appropriate antibodies. Tubulin was used as a loading control. Asterisk (*) in B marks Spn1-13Myc. The dashed vertical lines mark the positions of protein ladders which were excised out.

**Video Legends**

**Video 1. Accumulation of septin Spn1-mEGFP and the exocyst marked by Exo70-tdTomato to the division site.** The cell (strain JW9170) was imaged on a single-focal plane at the cell surface every 10 s in time-lapse TIRF microscopy (Nikon Ti Microscope). DIC image shows the cell had no septa at the beginning of the movie. Scale bar, 5 µm. Display rate: 10 frames per second (fps).

**Video 2. Dynamic localization of Exo70-tdTomato at the division site on a single-focal plane close to the cell surface.** The cell (strain JW9170) was imaged without delay (500 ms exposure) in time-lapse TIRF microscopy (Nikon Ti Microscope). DIC image shows the cell had no septa at the beginning of the movie. Scale bar, 5 µm. Display rate: 10 fps.

**Video 3. Dynamic localization of Exo70-tdTomato at the division site on the middle-focal plane.** The cell (strain JW9170) was imaged without delay (500 ms exposure) in time-lapse microscopy (Nikon Ti Microscope). DIC image shows the cell had no septa at the beginning of the movie. Scale bar, 5 µm. Display rate: 5 fps.

**Video 4. The predicted 3D structural model of *S. pombe* exocyst complex by AlphaFold3, highlighting the residues that interact with septins.** Individual subunits are colored distinctly and labeled. Surface exposed residues previously predicted as putative septin-interacting sites are highlighted in yellow.

**Video 5. The predicted 3D structural model of *S. pombe* septin octameric complex by AlphaFold3, highlighting the exocyst-interacting residues.** Two subunits of each septin Spn1 to Spn4 are used to construct the octameric complex. Different subunits are colored distinctly and labeled. Surface exposed residues previously predicted as putative exocyst-interacting sites are highlighted in yellow.

**Video 6. The predicted 3D structural model of *S. pombe* septins Spn1, Spn2, and Spn4 hexameric complex by AlphaFold3, highlighting the exocyst-interacting residues.** Two subunits of each Spn1, Spn2, and Spn4 are used to construct the hexameric complex. Different

bioRxiv preprint doi: https://doi.org/10.1101/2024.07.09.602728; this version posted July 1, 2025. The copyright holder for this preprint (which was not certified by peer review) is the author/funder, who has granted bioRxiv a license to display the preprint in perpetuity. It is made available under aCC-BY 4.0 International license.

subunits are colored distinctly and labeled. Surface exposed residues previously predicted as putative exocyst-interacting sites are highlighted in yellow.

**Video 7. The predicted 3D structural model of *S. pombe* septins Spn1, Spn3, and Spn4 hexameric complex by AlphaFold3, highlighting the exocyst-interacting residues.** Two subunits of each Spn1, Spn3, and Spn4 are used to construct the hexameric complex. Different subunits are colored distinctly and labeled. Surface exposed residues previously predicted as putative exocyst-interacting sites are highlighted in yellow.

**Video 8. Localization of Sec15 as a ring at the rim of the division plane during cytokinesis in WT cells.** Sec15-mEGFP cells (strain JW9726) were imaged at 3 min interval for 2 h in time-lapse confocal microscopy (UltraVIEW Vox CSUX1; PerkinElmer). 3D projections of fluorescence images from 14 slices spaced at 0.4 μm at each time point are shown. Display rate: 50 fps.

**Video 9. Mislocalization of Sec15 as a disk on division site in *spn1Δ* cells.** *sec15-mEGFP spn1Δ* cells (strain JW9852) were imaged at 3 min interval for 3 h in time-lapse confocal microscopy (UltraVIEW Vox CSUX1; PerkinElmer). 3D projections of fluorescence images from 14 slices spaced at 0.4 μm at each time point are shown. Display rate: 50 fps.

**Video 10. Mislocalization of Sec15 as a disk on division site in *spn4Δ* cells.** *sec15-mEGFP spn4Δ* cells (strain JW9853) were imaged at 3 min interval for 3 h in time-lapse confocal microscopy (UltraVIEW Vox CSUX1; PerkinElmer). 3D projections of fluorescence images from 14 slices spaced at 0.4 μm at each time point are shown. Display rate: 50 fps.

bioRxiv preprint doi: https://doi.org/10.1101/2024.07.09.602728; this version posted July 1, 2025. The copyright holder for this preprint (which was not certified by peer review) is the author/funder, who has granted bioRxiv a license to display the preprint in perpetuity. It is made available under aCC-BY 4.0 International license.

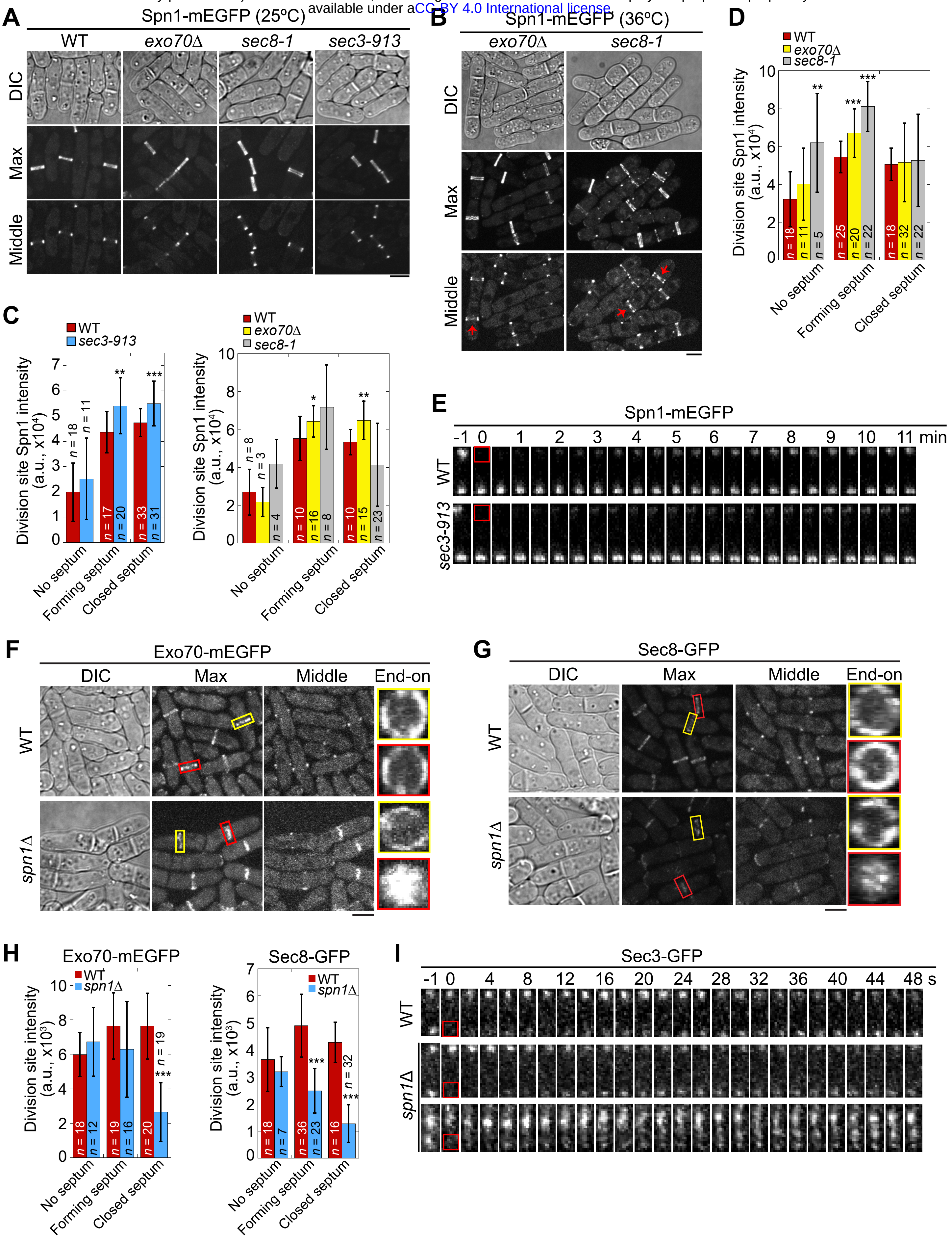


Supp Figure 1

bioRxiv preprint doi: https://doi.org/10.1101/2024.07.09.602728; this version posted July 1, 2025. The copyright holder for this preprint (which was not certified by peer review) is the author/funder, who has granted bioRxiv a license to display the preprint in perpetuity. It is made available under aCC-BY 4.0 International license.

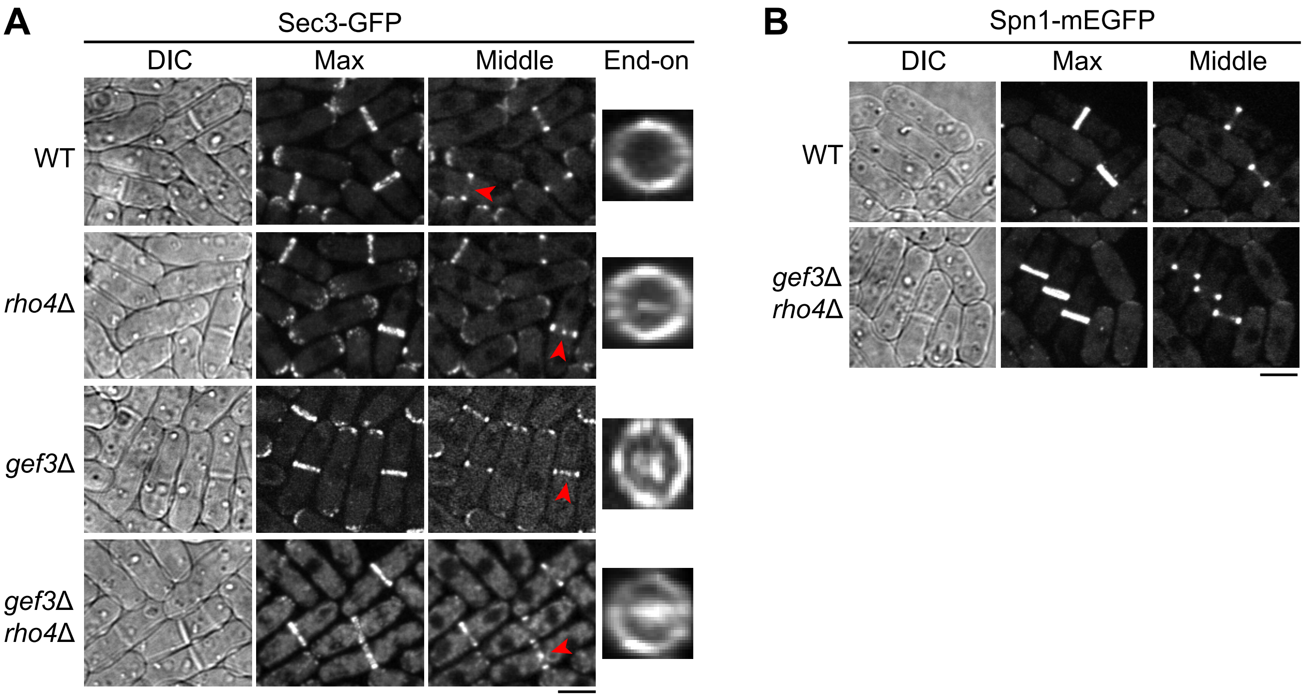


**Supp Figure 2**

bioRxiv preprint doi: https://doi.org/10.1101/2024.07.09.602728; this version posted July 1, 2025. The copyright holder for this preprint (which was not certified by peer review) is the author/funder, who has granted bioRxiv a license to display the preprint in perpetuity. It is made available under aCC-BY 4.0 International license.

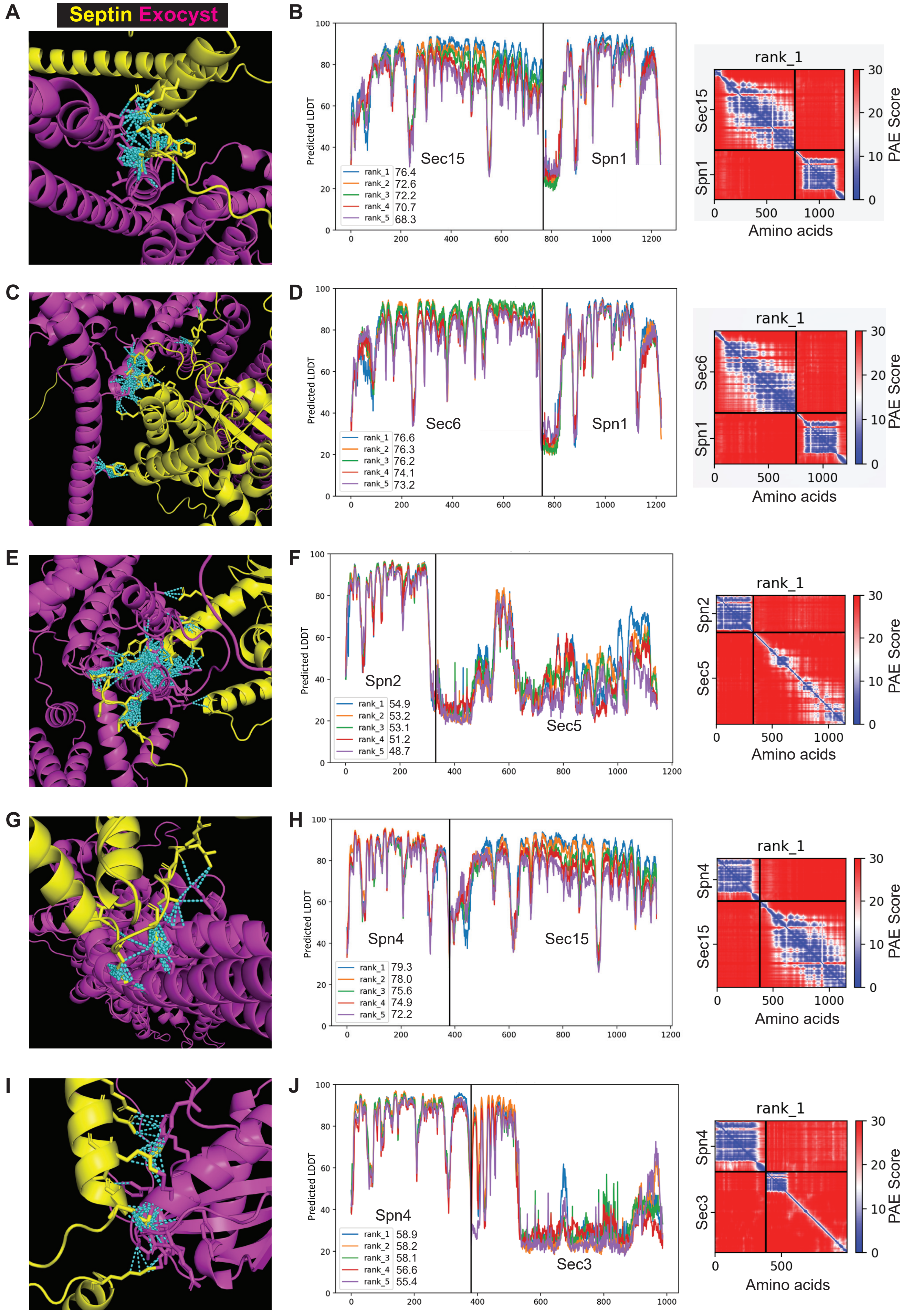


**Supp Figure 3**

bioRxiv preprint doi: https://doi.org/10.1101/2024.07.09.602728; this version posted July 1, 2025. The copyright holder for this preprint (which was not certified by peer review) is the author/funder, who has granted bioRxiv a license to display the preprint in perpetuity. It is made available under aCC-BY 4.0 International license.

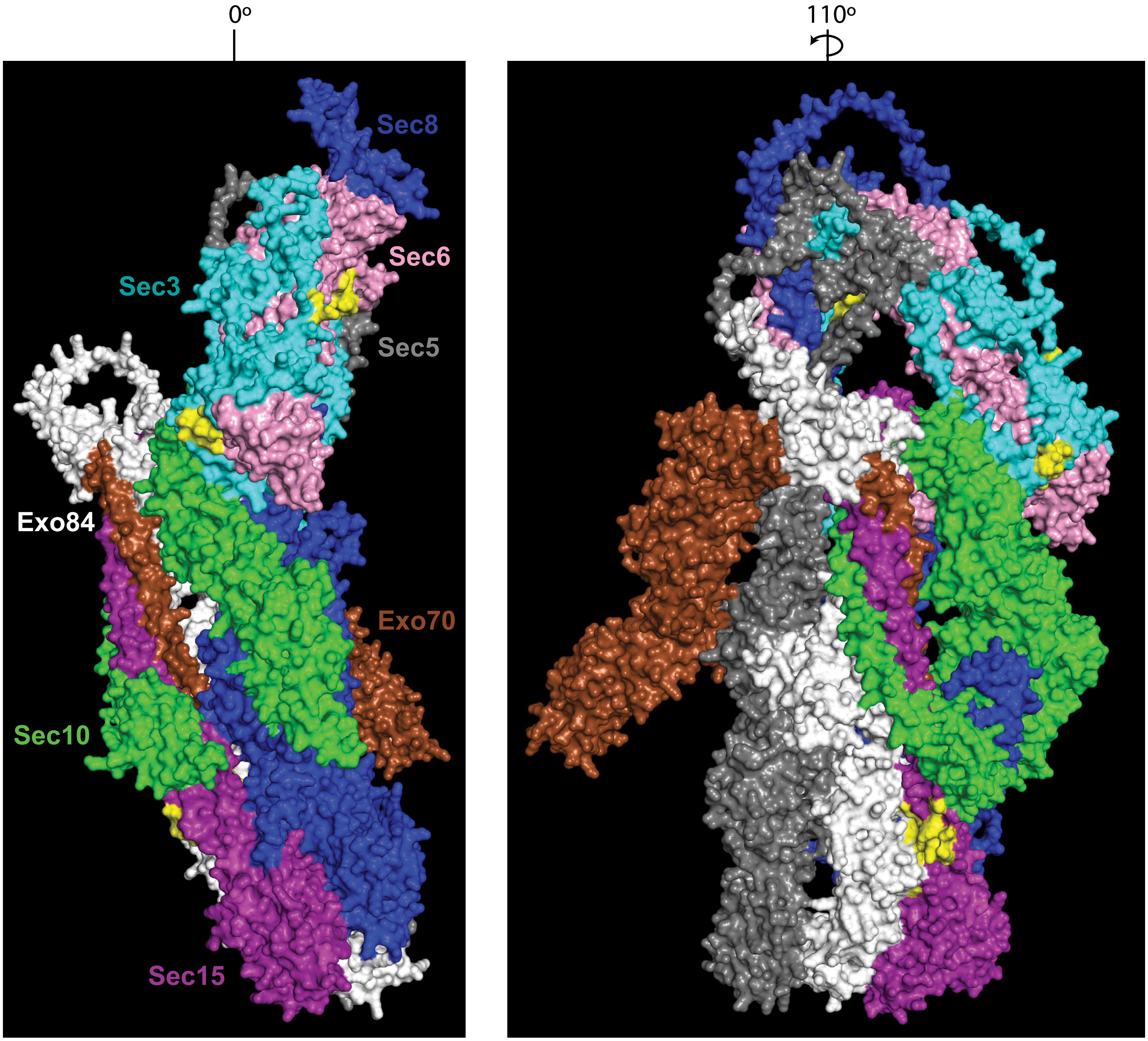


Supp Figure 4

bioRxiv preprint doi: https://doi.org/10.1101/2024.07.09.602728; this version posted July 1, 2025. The copyright holder for this preprint (which was not certified by peer review) is the author/funder, who has granted bioRxiv a license to display the preprint in perpetuity. It is made available under aCC-BY 4.0 International license.

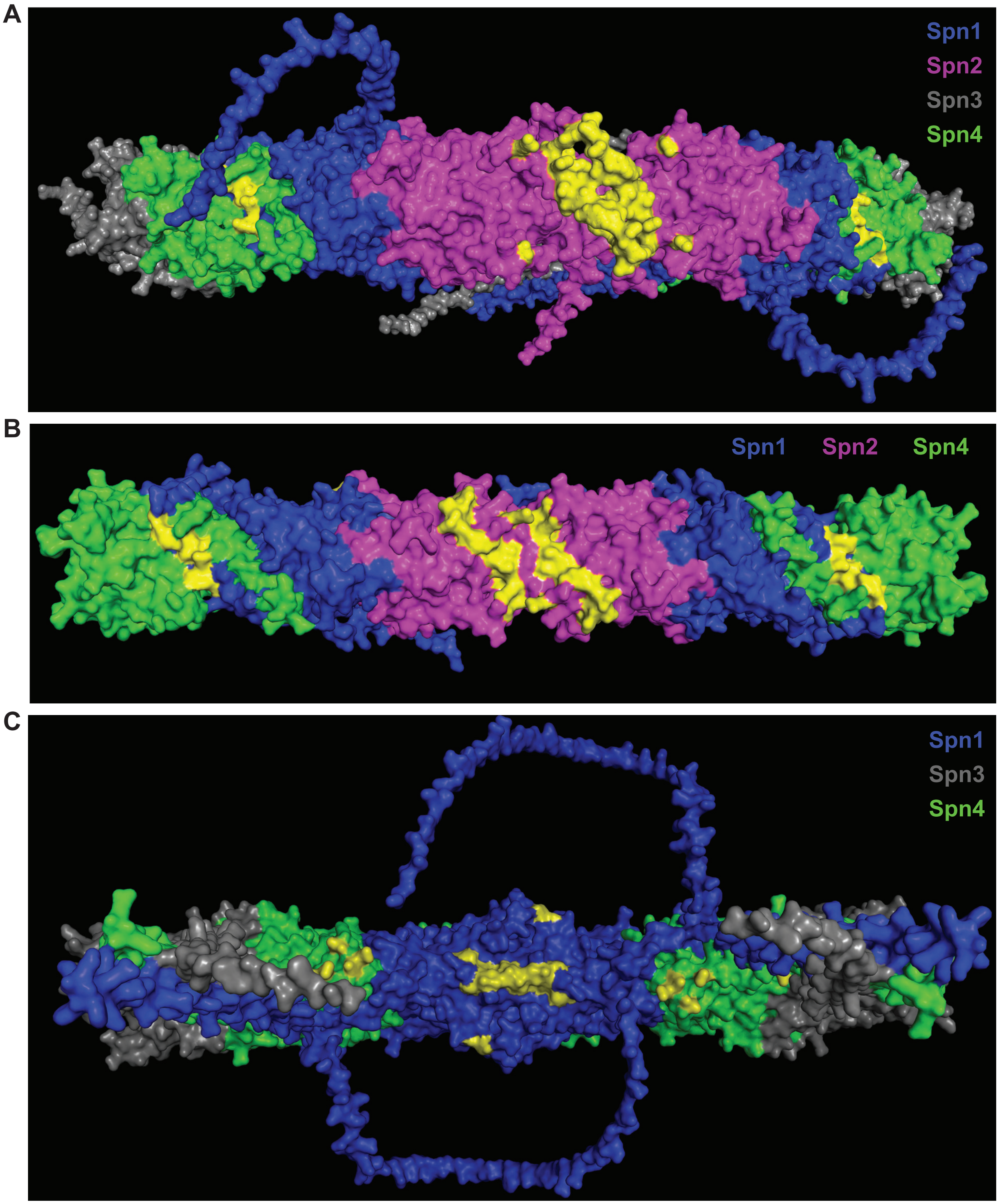


**Supp Figure 5**

bioRxiv preprint doi: https://doi.org/10.1101/2024.07.09.602728; this version posted July 1, 2025. The copyright holder for this preprint (which was not certified by peer review) is the author/funder, who has granted bioRxiv a license to display the preprint in perpetuity. It is made available under aCC-BY 4.0 International license.

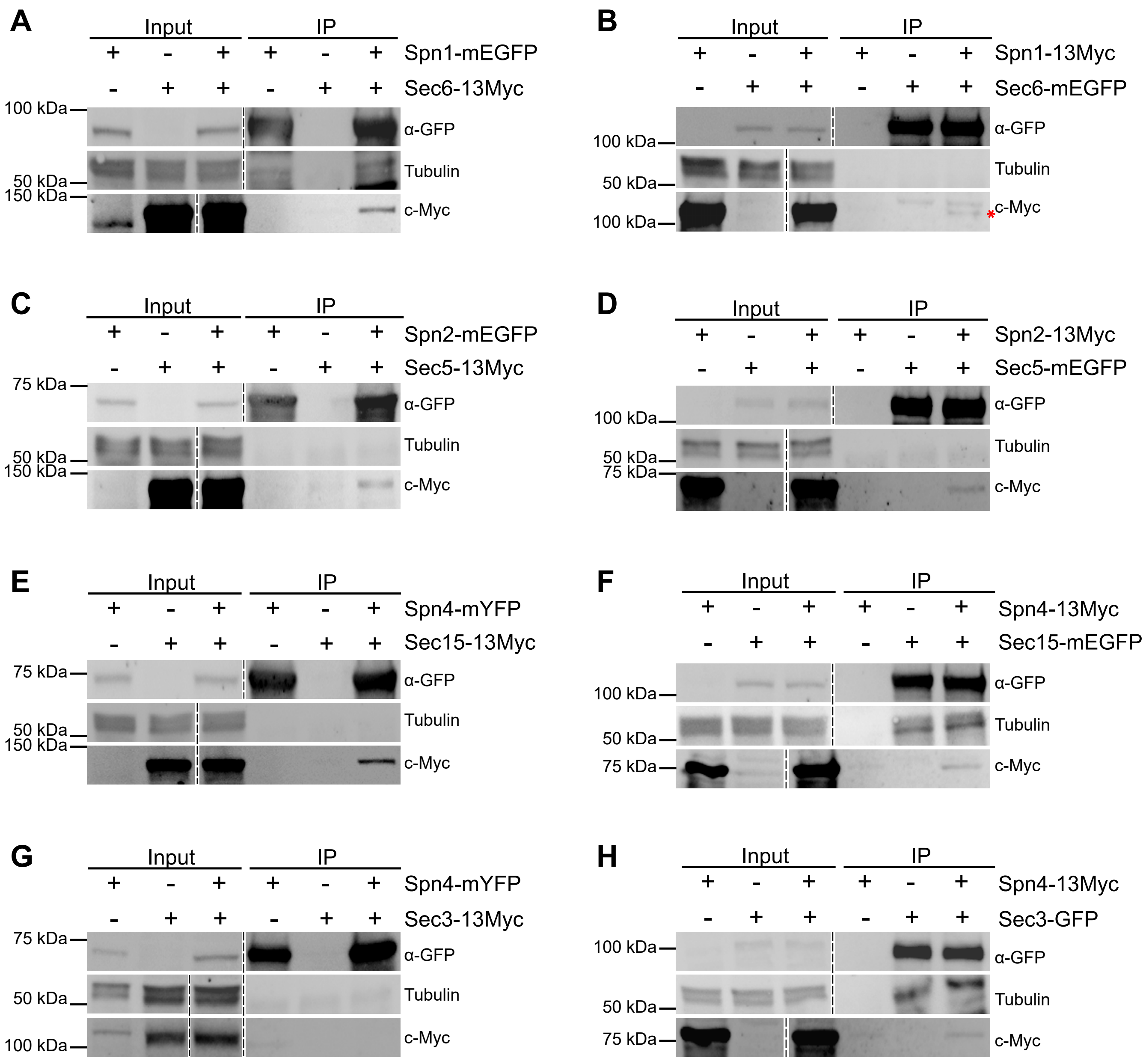


**Supp Figure 6**
