# Supplementary material for: Septins function in exocytosis via physical interactions with the exocyst complex in fission yeast cytokinesis
Source: bioRxiv. 2024 Jul 12:2024.07.09.602728. Preprint. [Version 1] doi: 10.1101/2024.07.09.602728 (PMC11257574; doi:10.1101/2024.07.09.602728)
Supplement: Supplementary file 1 [file NIHPP2024.07.09.602728v1-supplement-1.pdf]

bioRxiv preprint doi: https://doi.org/10.1101/2024.07.09.602728; this version posted July 12, 2024. The copyright holder for this preprint (which was not certified by peer review) is the author/funder, who has granted bioRxiv a license to display the preprint in perpetuity. It is made available under aCC-BY 4.0 International license.

Figure S1. Localization and division site levels of septin and exocyst subunits in mutants; and FRAP analyses of Spn1 and Sec3. (A and B) Localization of Spn1 in WT and exocyst mutants at 25°C (A) and 4 h at 36°C (B). Arrows indicate cells with mislocalized Spn1 at the center of the division plane. **(C and D)** Quantifications of Spn1 intensities at the division site in WT and exocyst mutants at 25°C (C) and 4 h at 36°C (D). No septum: cells with Spn1 signal at the division site but no septum is visible under DIC; forming septum: septum with a visible gap in the middle; closed septum: no visible gap in the septum. *, $P < 0.05$; **, $P < 0.01$; ***, $P < 0.001$. **(E)** FRAP analyses of Spn1 at the division site in WT and *sec3-913* cells grown at 36°C

bioRxiv preprint doi: https://doi.org/10.1101/2024.07.09.602728; this version posted July 12, 2024. The copyright holder for this preprint (which was not certified by peer review) is the author/funder, who has granted bioRxiv a license to display the preprint in perpetuity. It is made available under aCC-BY 4.0 International license.

for 4 h. Time-lapse images show recovery of Spn1 signals over time. Red box marks the region photobleached at time 0. **(F and G)** Localization of Exo70 (F) and Sec8 (G) in WT and *spn1*Δ cells. Yellow boxes, cells without a septum; Red boxes, cells with a closed septum. **(H)** Quantifications of Exo70 (left) and Sec8 (right) intensities at the division site in WT and *spn1*Δ cells. ***, $P < 0.001$. **(I)** FRAP analyses of Sec3 at the division site in WT and *spn1*Δ cells. Red box marks the region photobleached at time 0. Bars, 5 μm.

Figure S2. Sec3 and Spn1 localization in *gef3*, *rho4,* or *gef3 rho4* mutants. (A) Sec3 localization in WT, *rho4*Δ, *gef3*Δ, and *gef3*Δ *rho4*Δ cells. Arrowheads mark examples of the cells with mislocalized Sec3 at the center of the division plane in mutant but not WT cells. End-on views of the division plane of cells with a closed septum are shown on the last column. **(B)** Spn1 localization in WT and *gef3*Δ *rho4*Δ cells. Bars, 5 μm.

Figure S3. The 3D structural models of septin-exocyst interactions generated by AlphaFold. (A, C, E, G, I) Rank 1 model of AlphaFold2_advanced predicted interaction between Sec15 and Spn1 (A), Sec6 and Spn1 (C), Spn2 and Sec5 (E), Spn4 and Sec15 (G), and Spn4 and Sec3 (I). Septin subunits are colored in yellow and exocyst in magenta, contacts between interface residues with distance < 4 Å are colored in cyan**. (B, D, F, H, J)** pLDDT scores of five predicted models for Sec15 and Spn1 (B), Sec6 and Spn1 (D), Spn2 and Sec5 (F), Spn4 and Sec15 (H), and Spn4 and Sec3 (J).

Figure S4. Septins and exocyst interact physically. Reciprocal co-immunoprecipitation between Spn1 with Sec6 **(A, B)**; Spn2 with Sec5 **(C, D)**; Spn4 with Sec15 **(E, F)**; and Spn4 with Sec3 **(G, H)**. Septin or exocyst subunits tagged with mEGFP, GFP, mYFP, or 13Myc were immunoprecipitated, separated on SDS-PAGE, and incubated with appropriate antibodies. Tagged proteins were detected on iBright Imager. Tubulin was used as a loading control.

bioRxiv preprint doi: https://doi.org/10.1101/2024.07.09.602728; this version posted July 12, 2024. The copyright holder for this preprint (which was not certified by peer review) is the author/funder, who has granted bioRxiv a license to display the preprint in perpetuity. It is made available under aCC-BY 4.0 International license.

Asterisk (*) in B marks Spn1-13Myc. The dashed vertical lines mark the positions of protein ladders which was excised out.

Video Legends

Video 1. Accumulation of septin Spn1-mEGFP and the exocyst marked by Exo70-tdTomato to the division site. The cell (strain JW9170) was imaged on a single-focal plane at the cell surface every 10 s in time-lapse TIRF microscopy (Nikon Ti Microscope). DIC image shows the cell had no septa at the beginning of the movie. Scale bar, 5 µm. Display rate: 10 frames per second (fps).

Video 2. Dynamic localization of Exo70-tdTomato at the division site on a single-focal plane close to the cell surface. The cell (strain JW9170) was imaged without delay (500 ms exposure) in time-lapse TIRF microscopy (Nikon Ti Microscope). DIC image shows the cell had no septa at the beginning of the movie. Scale bar, 5 µm. Display rate: 10 fps.

Video 3. Dynamic localization of Exo70-tdTomato at the division site on the middle-focal plane. The cell (strain JW9170) was imaged without delay (500 ms exposure) in time-lapse microscopy (Nikon Ti Microscope). DIC image shows the cell had no septa at the beginning of the movie. Scale bar, 5 µm. Display rate: 5 fps.

Video 4. Localization of Sec15 as a ring at the rim of the division plane during cytokinesis in WT cells. Sec15-mEGFP cells (strain JW9726) were imaged at 3 min interval for 2 h in time-lapse confocal microscopy (UltraVIEW Vox CSUX1; PerkinElmer). 3D projections of fluorescence images from 14 slices spaced at 0.4 µm at each time point are shown. Display rate: 50 fps.

Video 5. Mislocalization of Sec15 as a disk on division site in *spn1Δ* cells.

bioRxiv preprint doi: https://doi.org/10.1101/2024.07.09.602728; this version posted July 12, 2024. The copyright holder for this preprint (which was not certified by peer review) is the author/funder, who has granted bioRxiv a license to display the preprint in perpetuity. It is made available under aCC-BY 4.0 International license.

sec15-mEGFP spn1Δ cells (strain JW9852) were imaged at 3 min interval for 3 h in time-lapse confocal microscopy (UltraVIEW Vox CSUX1; PerkinElmer). 3D projections of fluorescence images from 14 slices spaced at 0.4 μm at each time point are shown. Display rate: 50 fps.

Video 6. Mislocalization of Sec15 as a disk on division site in *spn4Δ* cells.

sec15-mEGFP spn4Δ cells (strain JW9853) were imaged at 3 min interval for 3 h in time-lapse confocal microscopy (UltraVIEW Vox CSUX1; PerkinElmer). 3D projections of fluorescence images from 14 slices spaced at 0.4 μm at each time point are shown. Display rate: 50 fps.

bioRxiv preprint doi: https://doi.org/10.1101/2024.07.09.602728; this version posted July 12, 2024. The copyright holder for this preprint (which was not certified by peer review) is the author/funder, who has granted bioRxiv a license to display the preprint in perpetuity. It is made available under aCC-BY 4.0 International license.

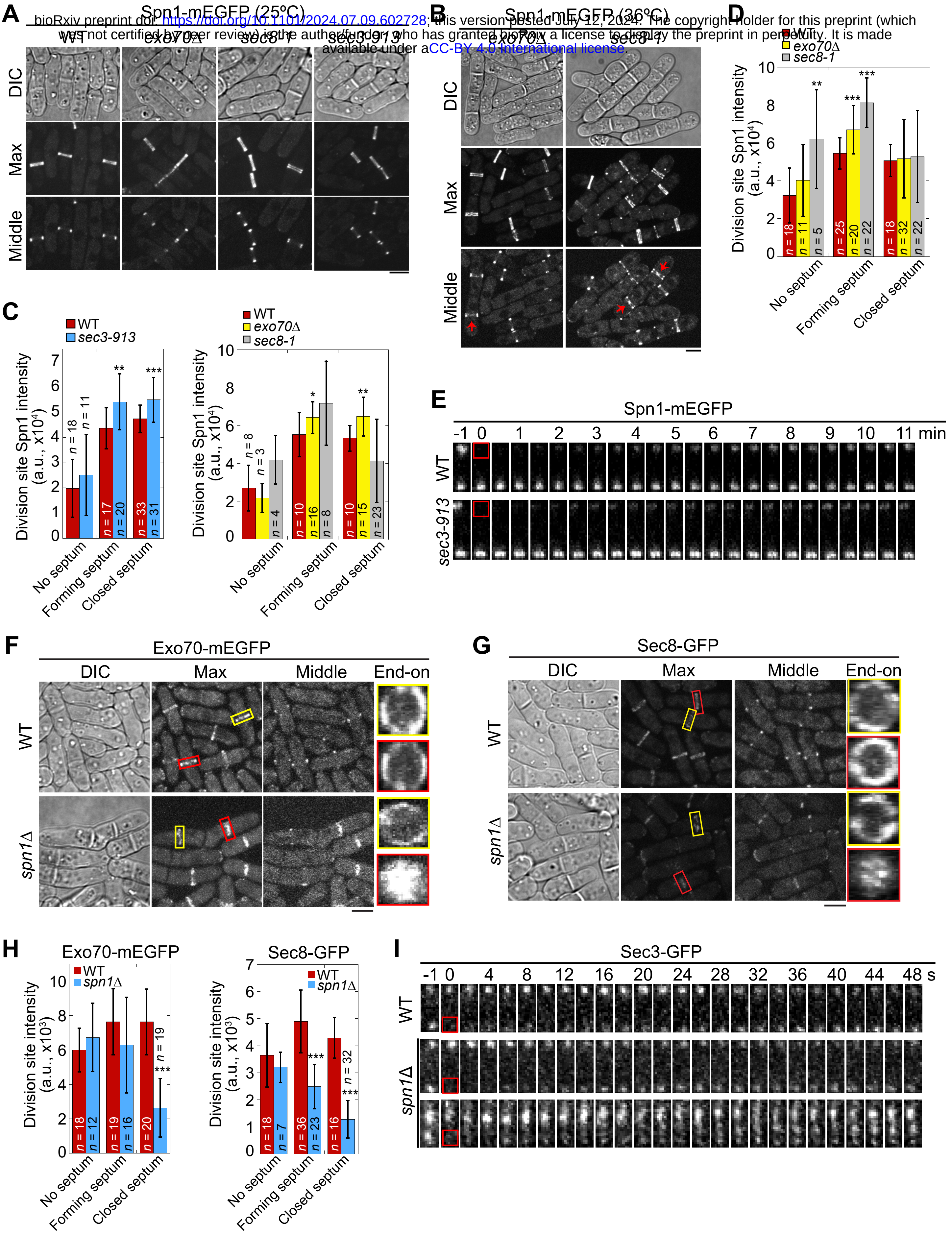

Supp Figure 1

bioRxiv preprint doi: https://doi.org/10.1101/2024.07.09.602728; this version posted July 12, 2024. The copyright holder for this preprint (which was not certified by peer review) is the author/funder, who has granted bioRxiv a license to display the preprint in perpetuity. It is made available under aCC-BY 4.0 International license.

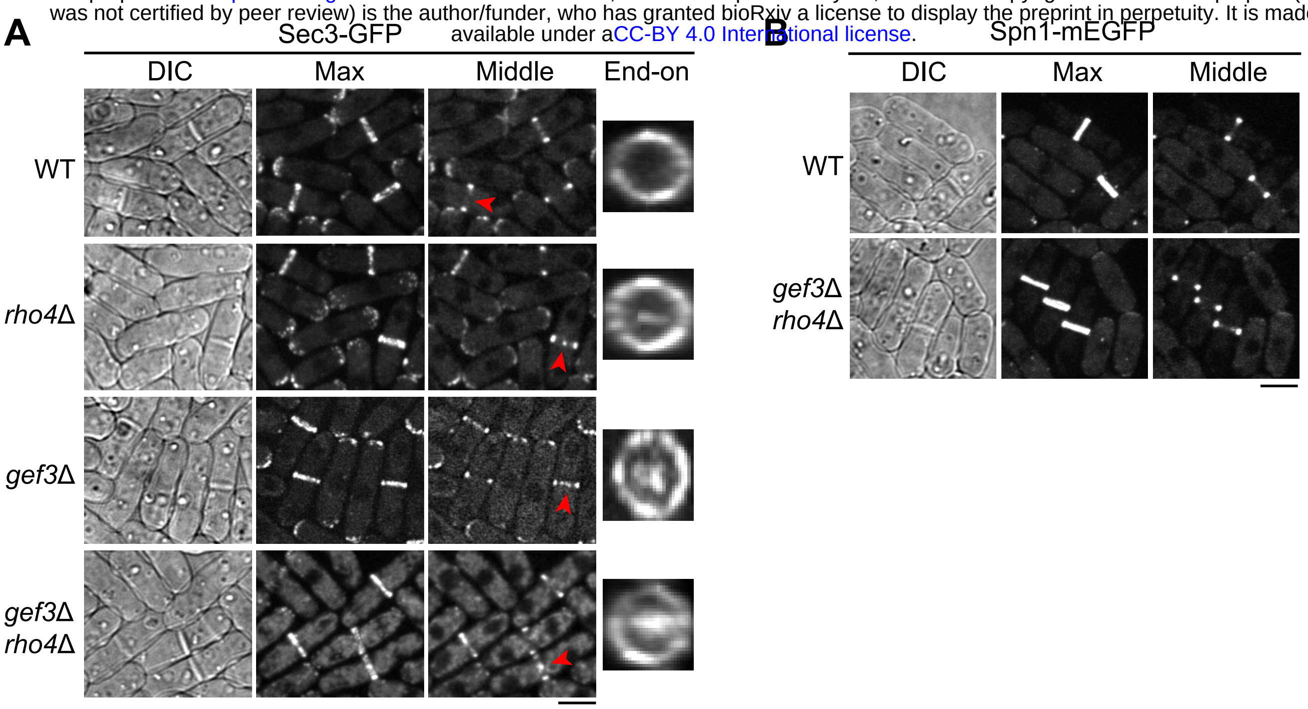

Supp Figure 2

bioRxiv preprint doi: https://doi.org/10.1101/2024.07.09.602728; this version posted July 12, 2024. The copyright holder for this preprint (which was not certified by peer review) is the author/funder, who has granted bioRxiv a license to display the preprint in perpetuity. It is made available under aCC-BY 4.0 International license.

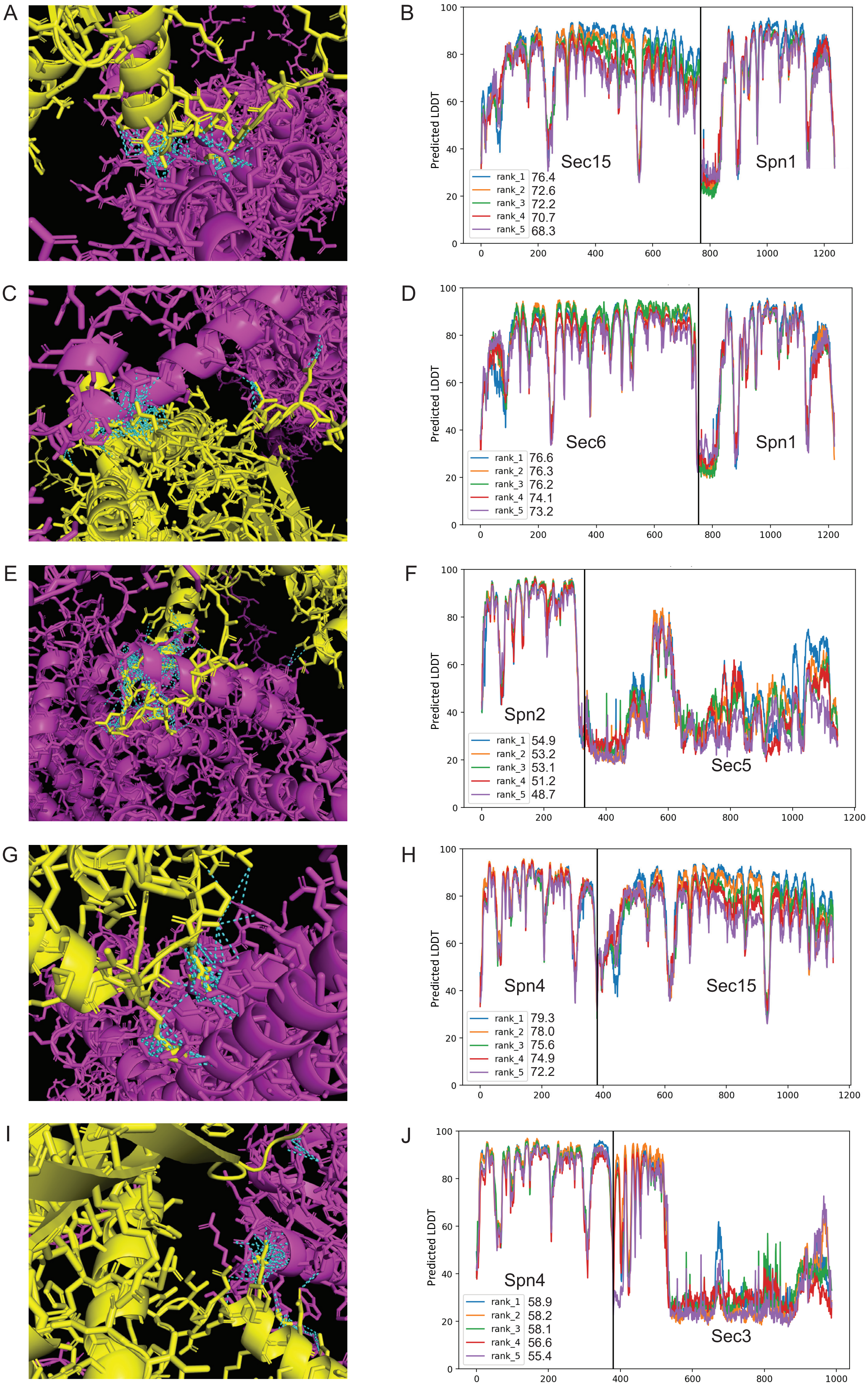

Supp Figure 3

bioRxiv preprint doi: https://doi.org/10.1101/2024.07.09.602728; this version posted July 12, 2024. The copyright holder for this preprint (which was not certified by peer review) is the author/funder, who has granted bioRxiv a license to display the preprint in perpetuity. It is made available under aCC-BY 4.0 International license.

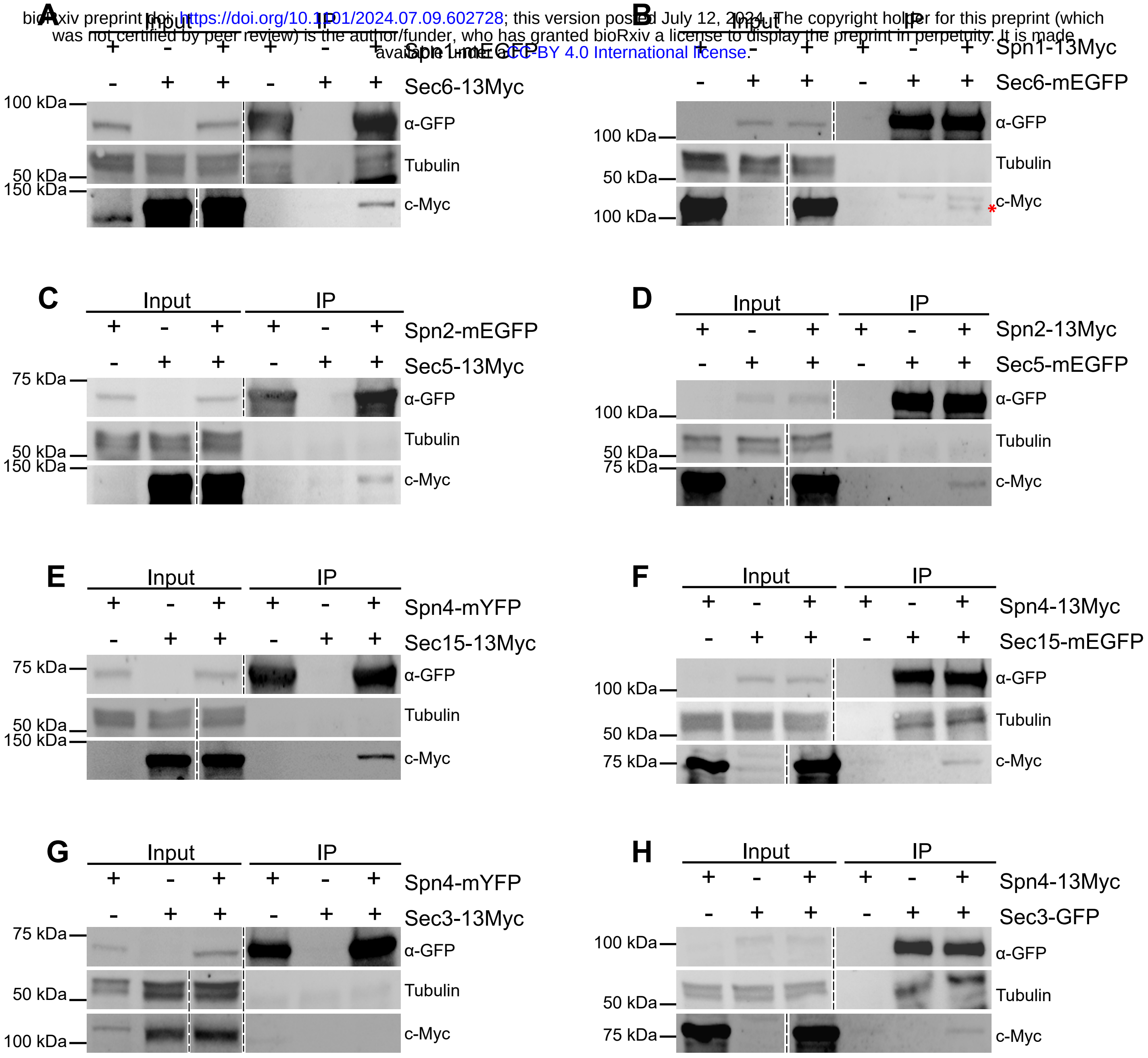

Supp Figure 4
